# Endometrial microstimulation effects on endometrial receptivity assessed by transvaginal color Doppler sonography

**DOI:** 10.1186/s12905-022-02096-z

**Published:** 2022-12-09

**Authors:** Fang Cheng, Bao-Mei Xv, Yan-Lin Liu, Rui Sun, Lin Wang, Jin-Ling Yi

**Affiliations:** 1 Department of Obstetrics, The Fifth Affiliated Hospital of Xinjiang Medical University, Urumqi, 830011 China; 2 Department of Gynecology, The Fifth Affiliated Hospital of Xinjiang Medical University, No.118 of West Henan Road, Urumqi, 830011 China; 3 Department of Ultrasonic Diagnosis, The Fifth Affiliated Hospital of Xinjiang Medical University, Urumqi, 830011 China

**Keywords:** Endometrial receptivity, Endometrial microstimulation, Transvaginal color Doppler sonography, Implantation window phase

## Abstract

**Objective:**

This study investigated the effect of endometrial microstimulation (EM) on endometrial receptivity using transvaginal color Doppler sonography (TVCDS).

**Method:**

Women of childbearing age who were preparing to conceive (*n* = 90) were randomly divided into the EM group (*n* = 30), who were examined by EM on days 3–5 of the menstrual cycle, and the control group (*n* = 60). TVCDS was conducted during the implantation window phase, and endometrial thickness, endometrial pattern, endometrial movement, blood flow type, and uterine and spiral arterial hemodynamic parameter measurements were made. The groups were compared to identify differences.

**Results:**

Endometrial thickness (0.97 ± 0.18 cm and 0.95 ± 0.17 cm), endometrial movement (type 1: 46.7% and 51.7%; type 2: 30.0% and 28.3%; type 3: 6.7% and 5.0%; type 5: 16.7% and 15.0%), and hemodynamic parameters of the uterine (pulsatility index [PI]: 2.46 ± 0.50 and 2.41 ± 0.48; resistance index [RI]: 0.85 ± 0.05 and 0.84 ± 0.05) and spiral (PI: 1.11 ± 0.32 and 1.19 ± 0.33; RI: 0.48 ± 0.11 and 0.51 ± 0.08) arteries did not differ significantly between groups (*P* > 0.05). However, the endometrial pattern (a trilaminar pattern: 80.0% and 58.3%; *P* = 0.041) and blood flow type (type I: 16.7% and 43.3%; type II: 63.3% and 40.0%; type III 20.0% and 16.7%; *P* = 0.038) differed significantly between groups.

**Conclusion:**

Endometrial microstimulation did not alter endometrial pathological staging, endometrial thickness, or movement, nor did it affect uterine and spiral arterial blood flow parameters. However, it may be able to abrade abnormal endometrial tissue, optimizing the endometrial pattern. Endometrial microstimulation may support local spiral artery regeneration and increase endometrial blood supply in new cycles.

## Introduction

Endometrial receptivity directly impacts conception, with nearly 70% of implantation failures due to poor receptivity [1]. Days 20–23 of the normal menstrual cycle, reflecting the middle of the secretory phase (i.e., days 6–9 after ovulation and 7 days after peak luteinizing hormone [LH] levels), is known as the implantation window phase when the endometrium is most receptive. Transvaginal color Doppler sonography (TVCDS) can be used to evaluate endometrial receptivity [2, 3]. TVCDS evaluation parameters include endometrial thickness, endometrial pattern, endometrial movement, and uterine artery and sub-endometrial blood flow [4].

Endometrial microstimulation, intrauterine perfusion, and hysteroscopy can improve endometrial receptivity. However, it is difficult to determine which method is more suitable for endometrial preparation. Since most previous studies were retrospective, their comparisons may be biased. Moreover, conducting prospective randomized controlled studies with large samples is imperative, which may provide more valuable data.

Some studies have shown that abrading the endothelium may support local cell regeneration, stimulate spiral artery regeneration, and improve spiral artery function, increasing the local blood supply and facilitating egg implantation. Therefore, this study investigated the value of endometrial microstimulation (EM) in endometrial receptivity using sonographic imaging of the uterus during the implantation window phase. Its findings will guide some infertile women of childbearing age to become pregnant successfully.

## Materials and methods

### Materials

#### Participants

This study included 90 women of childbearing age who were preparing to conceive and had visited the Department of Gynecology at the Fifth Affiliated Hospital of Xinjiang Medical University (China) between May 2019 and September 2021. The participants were randomly divided into two groups: the EM group (*n* = 30), who received EM on days 3–5 of the menstrual cycle, and a TVCDS examination and endometrial biopsy during the implantation window phase (days 6–9 after ovulation); the control group (*n* = 60), who only received a TVCDS examination during the implantation window phase. In addition, 30 participants were randomly selected from the control group (biopsy subgroup) to undergo endometrial biopsy.

#### Inclusion criteria

The study’s inclusion criteria were as follows: (1) aged 25–35 years with regular menstruation and a menstrual cycle length of 28 ± 2 days; (2) no organic gynecological or endocrine diseases or functional vascular disorders (e.g., diabetes mellitus and hypertension, ruled out by specialist examinations); (3) no history of hormone medication within the preceding 6 months; (4) no smoking history.

#### Exclusion criteria

Patients with infertility were excluded from this study.

### Methods

#### Endometrial microstimulation

Participants in the EM group had their endometrium circularly scraped with a Jingyou SPA-I Endocell endometrial cell sampler (Beijing Saipu Jiuzhou Science and Technology Development Co., Ltd.) on days 3–5 of their menstrual cycle. Following stringent sterilization, the sampler was slowly inserted until the head end reached the uterine fundus. Next, the sampler ring was released while the outer casing wand remained in place. Then, the sampler’s handle was rotated 3–5 turns clockwise. Next, the sampler ring was recovered while the outer casing remained unmoved. Finally, the handle was withdrawn to remove the sampler.

#### Detection time

Changes in the ovarian follicles were examined daily by TVCDS starting on day 10 of menstruation in both EM and control groups. When the follicle size was ≥ 14 mm, LH levels were tracked daily via a urine LH semi-quantitative test to determine the highest LH level and observe ovulation. TVCDS was performed on days 6–9 after ovulation, and the selected ultrasonographic indicators were measured. Then, an endometrial biopsy was taken by EM in the EM group and the control biopsy subgroup for pathological examination.

#### Ultrasonographic detection

A GE Logiq 5 color ultrasound diagnostic instrument was used with a probe frequency of 5–7 MHz, wall filtering < 100 Hz, and a pulse repetition frequency of 4–6 cm/s. The sampling volume was 1 mm, the umbrella expansion angle was 120°, and the angle between the blood flow direction and the sound speed was < 30°.

*Endometrial thickness* Complete endometrial images were acquired longitudinally. Endometrial thickness was measured as the distance between the endometrium’s strong echogenicity on both sides of the uterine fundus.

*Endometrial pattern* Two endometrial patterns were considered. A trilaminar pattern reflects strong echogenicity in the uterine cavity’s outer layer and midline. A homogeneous pattern reflects no midline echogenicity in the uterine cavity.

**Fig. 1:**
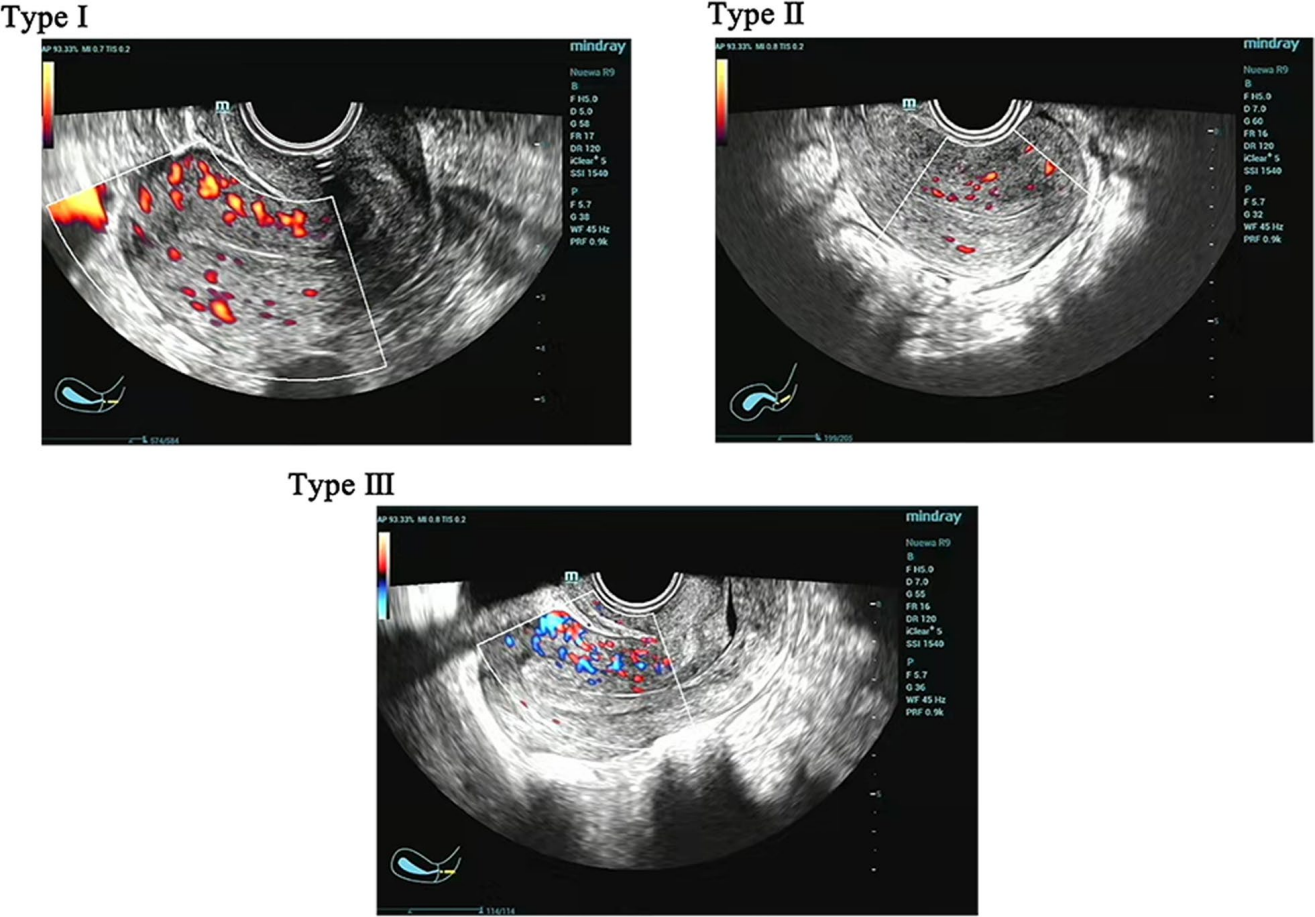
The color ultrasonography image of the type of endometrial blood flow

*Endometrial blood flow type* Type I had no blood flow reaching the endometrium’s outer edge. Type II had blood flow reaching the endometrium’s outer edge. Type III had blood flow reaching and penetrating the endometrium (Fig. 1).

*Endometrial movement* Five endometrial movement types [5] were observed. Type 1, where the endometrial movement begins at the cervix and points to the fundus. Type 2, where the movement begins at the fundus and points to the cervix. Type 3, where the movement begins concurrently from the endocervix and the fundus. Type 4, where movement spreads from different parts of the uterine cavity. Type 5, no obvious endometrial movement is observed.

*Uterine artery hemodynamics* Doppler signals in the uterine artery’s isthmus (bilateral) were acquired alongside recording a clear blood flow pulse spectrum. Five cardiac cycles were observed, and the best cross-section image was selected. The uterine artery pulsatility index (PI; the maximum systolic blood flow velocity [Vmax] minus the end-diastolic blood flow velocity) divided by the mean velocity [Vmean]) and resistance index (RI; Vmax minus the end-diastolic blood flow velocity) divided by Vmax) were calculated by the device.

*Spiral arterial hemodynamic status* The best endometrial spiral artery section was selected to clearly detect the pulsed Doppler flow spectrum and obtain the PI, RI, Vmax, and Vmean values.

All examinations were conducted by designated doctors to minimize inter-physician variation.

### Statistical methods

The SPSS Statistics 25.0 software was used to perform data analyses. The Chi-square test was used to compare count data. Measurement data are expressed as mean ± standard deviation and were compared using a *t*-test of variance. All results with *P* < 0.05 were considered statistically significant.

## Results

### General characteristic differences between groups

The two groups comprised 90 participants. Age, pregnancy history, and delivery history did not differ significantly between groups, and their data were comparable (Table 1).

**Table 1:** Comparison of the general characteristics

Group	The number of cases (case)	Age (year)	Pregnancy history (subject)	Delivery history (subject)
Group A	30	29.77 ± 2.75	21	6
Group B	60	29.38 ± 3.14	46	18

*P* > 0.05 for the comparison between the two groups

### Comparison of endometrial thickness, pattern, blood flow, and movement between groups

Endometrial thickness and movement did not differ significantly between groups. However, blood flow type did differ significantly between groups (*P* = 0.038). The trilaminar pattern was significantly more common in the EM (80.0%) group than in the control group (58.3%; *P* = 0.041; Table 2).

**Table 2:** Comparison of the endometrial thickness, pattern, and movement

Item	Group A [the number of cases (%)]	Group B [the number of cases (%)]
Endometrial thickness (cm)	0.97 ± 0.18	0.95 ± 0.17
Endometrial pattern:Trilaminar pattern	24 (80.0%)*	35 (58.3%)
Homogeneous pattern	6 (20.0%)	25 (41.7%)
Endometrial blood flow: Type I	5 (16.7%)**	26 (43.3%)
Type II	19 (63.3%)	24 (40.0%)
Type III	6 (20.0%)	10 (16.7%)
Endometrial movement: Type 1	14 (46.7%)	31 (51.7%)
Type 2	9 (30.0%)	17 (28.3%)
Type 3	2 (6.7%)	3 (5.0%)
Type 5	5 (16.7%)	9 (15.0%)

*Compare with group B, the difference was statistically significant, *P* = 0.041

** Compare with group B, the difference was statistically significant, *P* = 0.038

### Comparison of the PI, RI, Vmax, and Vmean of the uterine and spiral arteries between groups

The blood flow parameters of the uterine and spiral arteries did not differ significantly between groups (Table 3).

**Table 3:** Comparison of the blood flow parameters of the uterine artery and spiral artery

Type of artery	Blood flow parameter	Group A	Group B
The uterine artery	PI	2.46 ± 0.50	2.41 ± 0.48
	RI	0.85 ± 0.05	0.84 ± 0.05
	Vmax	30.59 ± 5.16	30.16 ± 5.82
	Vmean	4.45 ± 1.75	5.13 ± 1.41
The spiral artery	PI	1.11 ± 0.32	1.19 ± 0.33
	RI	0.48 ± 0.11	0.51 ± 0.08
	Vmax	6.46 ± 1.63	6.96 ± 1.70
	Vmean	3.26 ± 0.87	3.11 ± 0.75

*P* > 0.05 for the comparison between the two groups

### Comparison of endometrial pathological findings between groups

The endometrial pathological staging did not differ significantly between the EM group (four in the early, 23 in the middle, and three in the late secretion phases) and the control biopsy subgroup (six in the early, 20 in the middle, and four in the late secretion phases; Table 4).

**Table 4:** Comparison of the pathological findings

Group	The number of cases	The early secretion stage [The number of cases (%)]	The middle secretion stage [The number of cases (%)]	The late secretion stage [The number of cases (%)]
Group A	30	4 (13.3%)	23 (76.7%)	3 (10.0%)
Group B1	30	6 (20.0%)	20 (66.7%)	4 (13.3%)

*P* > 0.05 for the comparison between the two groups

## Discussion

### Endometrial receptivity and endometrial microstimulation

The implantation window phase determines fertilized egg implantation. There are several ways to improve the implantation rate, including hysteroscopic procedures, diagnostic curettage, and pharmacological cycle therapy [6, 7]. It is currently difficult to select an optimal method because bias cannot be avoided in many retrospective studies. Therefore, conducting additional prospective studies may result in more valuable data.

Scratching the endothelium may support local cell regeneration, stimulate spiral artery regeneration, and improve spiral artery function, increasing the local blood supply and promoting the expression of factors related to fertilized egg implantation. Moreover, scratching may remove abnormal endothelium and facilitate egg implantation [8].

Mechanical endometrium stimulation is currently attracting significant interest [9, 10]. In this study, circular EM (scratching) was performed using an endometrial cell sampler. This simple technique uses a moderate scratching depth to produce uniform endometrium stimulation without damaging its basal layer. Additionally, irregular endometrial tissue was removed completely from the uterine cavity with good participant compliance and no adverse reactions.

### Endometrial thickness, pattern, blood flow type, and movement

Several studies have shown the association between successful conception and endometrial thickness. However, others have reached the opposite conclusion [11]. This study found that EM did not significantly change endometrial thickness.

Most studies agree that different endometrial patterns lead to variable conception outcomes. A trilaminar endometrial pattern is typically more advantageous for successful implantation. This study’s results also showed that a trilaminar pattern was significantly more common in the EM group (80.0%) than in the control group (58.3%; *P* = 0.041). Therefore, EM might optimize the endothelial pattern and make it more conducive to implantation by scratching away abnormal endometrial tissue.

The association between clinical pregnancy rates and submucosal flow characterization is inconclusive. Theoretically, type III submucosal flow suggests that spiral arteries reach the endothelium. These arteries deliver a richer blood supply than types I and II. While an increased implantation rate was found [12] with increased sub-endometrial blood flow, the difference was not statistically significant. This study has shown that submucosal flow staging by ultrasonography is easy and feasible. Based on its results, submucosal flow staging type I was significantly less common in the EM group (16.7%) than in the control group (43.3%). In contrast, type II was significantly more common in the EM group (63.3%) than in the control group (40.0%). These differences were statistically significant (*P* = 0.038). Therefore, EM might assist in local spiral artery regeneration, increasing the endometrial blood supply in new cycles.

Sub-endometrial muscle layer movement manifests as endothelium movement under ultrasound and is associated with steroid hormones in women. Endometrium movement in healthy women of reproductive age shifts from pre-ovulatory type 2 to post-ovulatory type 1 and may prevent miscarriage. Frequent and abnormal movements may lead to conception failure. Consistent with these results, this study found a reduced endometrial type 2 movement (28.3%), while type 1 movement (51.7%) occurred most frequently after ovulation in healthy women of reproductive age. Therefore, EM did not alter endometrial movement.

### Uterine and spiral artery blood flow parameters

Blood flow in the uterine artery in healthy women of childbearing age generally shows corresponding cyclical changes to the menstrual cycle. For example, a PI < 2 was associated with an increased pregnancy rate [13], suggesting that uterine artery blood flow increases endometrial receptivity. In contrast, a PI > 3 was associated with a decreased pregnancy rate. While some studies [14] have shown that receptivity can be assessed using PI and RI, other studies contradict this view [15]. In this study, Pi and RI differences did not differ significantly between the EM and control groups (*P* > 0.05). Theoretically, it was also difficult to imagine that EM could affect the blood flow in the uterine artery.

The spiral arteries represent the uterine artery’s terminal branch. They are regulated by hormones and reflect an increased blood supply after ovulation, creating favorable conditions for conception. Therefore, spiral artery parameters can also assess the endometrium’s ability to receive fertilized eggs [16]. This study showed that blood supply to the spiral arteries did not differ significantly between the EM and control groups (*P* > 0.05). Theoretically, EM will positively affect the endometrial blood supply. However, this effect may not be sufficient to change these parameters.

The uterine artery’s terminal branches are slender and curved, with slow blood flow. Additionally, some equipment may have limitations that make clinical measurements more difficult. This study did not detect a spiral arterial signal in nine participants. Few studies have explored EM’s impact on subendothelial blood flow and receptivity. Nevertheless, the significance of the presence of endothelial spiral arteries underscores the value of this study.

### Endometrial pathological staging

Based on morphological observations, pathological staging did not differ significantly between this study’s EM and control groups (*P* > 0.05), indicating that EM did not affect endometrial staging. Pathological staging may be too simplistic to study endometrial receptivity. However, this study suggests that TVCDS combined with urine LH semi-quantitative test tracking may effectively monitor ovulation and determine the implantation window phase. When endometrial pathology is not required, non-invasive ultrasonography may be used as a single method for meeting clinical needs.

This study showed that EM might alter the uterine epithelium’s ability to admit fertilized eggs by influencing the endometrial pattern and sub-endometrial blood flow type. While few studies have used TVCDS to predict the uterine epithelium’s ability to accept fertilized eggs, this non-invasive and convenient method may be one of the best approaches for predicting endometrial receptivity.

## Conclusions

This study’s primary conclusions are as follows.

1. EM did not alter endometrial pathological staging, thickness, or movement, nor did it affect the blood flow parameters of the uterine and spiral arteries.
2. EM may be able to abrade abnormal endometrial tissue, optimizing the endometrial pattern.
3. EM might support local spiral arteries to regenerate, increasing the endometrial blood supply in subsequent cycles.
4. EM might improve endometrial receptivity by affecting the endometrial pattern and sub-endometrial blood flow type.

## Data Availability

All data generated or analysed during this study are included in this article. Further enquiries can be directed to the corresponding author.

## References

[1] Shiono Y, Watanabe K, Hosogane N (2012). Sterility of posterior elements of the spine in posterior correction surgery. Spine (Phila Pa 1976).

[2] Morad AWA, Farag MAE (2015). Impact of letrozole on ultrasonographic markers of endometrial receptivity in polycystic ovary syndrome women with poor endometrial response to clomiphene citrate despite adequate ovulation. Midd East Fertil Soc J.

[3] Xu XH, Chen YC, Xu YL, Feng ZL, Liu QY, Guo X (2021). Garcinone E blocks autophagy through lysosomal functional destruction in ovarian cancer cells. World J Tradit Chin Med.

[4] Altmäe S, Mendoza-Tesarik R, Mendoza C (2018). Effect of growth hormone on uterine receptivity in women with repeated implantation failure in an oocyte donation program: a randomized controlled trial. J Endocr Soc.

[5] Cheng F, Li T, Wang QL (2015). Effects of hydrosalpinx on ultrasonographic parameters for endometrial receptivity during the window of implantation measured by power color Doppler ultrasound. Int J Clin Exp Med.

[6] Gong H, He B, Zhou H (2020). Progress in treatment of endometrial receptivity. J Liaoning Univ Tradit Chin Med.

[7] Zhang TH, Liang JX, Long DL, Ma M, Chen LG, Lu DX (2022). Tocolysis effects of traditional Chinese medicine and their effective components. World J Tradit Chin Med.

[8] Lv Z, Sun W, Ni Q (2015). Effectiveness of local mild curettage on improving endometrium receptivity: a meta-analysis. Chin J Woman Child Health Res.

[9] Kitaya K, Matsubayashi H, Takaya Y (2016). Clinical background affecting pregnancy outcome following local endometrial injury in infertile patients with repeated implantation failure. Gynecol Endocrinol.

[10] Sa Y, Sun Z (2018). Clinical study of improving endometrial receptivity by endometrial debridement. Chin J Birth Health Hered.

[11] Kasius A, Smit JG, Torrance HL (2014). Endometrial thickness and pregnancy rates after IVF: a systematic review and Metaanalysis. Hum Reprod Update.

[12] Khonelidze NL, Tsagareishvili GG, Koiava MA (2005). The role of ultrasound scanning of endometrium in the superovulation stimulation program during in vitro fertilization and embryo transfer. Georgian Med News.

[13] Gunther V, Waldvogel D, Nosswitz M (2012). Dissection of Drosophila MTF-1 reveals a domain for differential target gene activation upon copper overload vs. copper starvation. Int J Bioehem Cell Biol.

[14] Hou J, Wang X, Lu P (2021). Correlation between the level of immune cells in peripheral blood and endometrial receptivity. Maternal Child Health Care China.

[15] Chen H, Jiang M (2017). Ultrasonic evaluation of endometrial receptivity. J Diagn Concepts Pract.

[16] Li T, Hu J, He GH (2012). Up-regulation of NDRG2 through nuclear factor-kappa B is required for Leydig cell apoptosis in both human and routine infertile testes. Biochim Biophys Acta.

